# Cryptic relatedness in epidemiologic collections accessed for genetic association studies: experiences from the Epidemiologic Architecture for Genes Linked to Environment (EAGLE) study and the National Health and Nutrition Examination Surveys (NHANES)

**DOI:** 10.3389/fgene.2015.00317

**Published:** 2015-10-26

**Authors:** Jennifer Malinowski, Robert Goodloe, Kristin Brown-Gentry, Dana C. Crawford

**Affiliations:** ^1^Center for Human Genetics Research, Vanderbilt University, NashvilleTN, USA; ^2^Department of Epidemiology and Biostatistics, Institute for Computational Biology, Case Western Reserve University, ClevelandOH, USA

**Keywords:** genetic epidemiology, epidemiology, cross-sectional, NHANES, cryptic relatedness, genetic association study, EAGLE

## Abstract

Epidemiologic collections have been a major resource for genotype–phenotype studies of complex disease given their large sample size, racial/ethnic diversity, and breadth and depth of phenotypes, traits, and exposures. A major disadvantage of these collections is they often survey households and communities without collecting extensive pedigree data. Failure to account for substantial relatedness can lead to inflated estimates and spurious associations. To examine the extent of cryptic relatedness in an epidemiologic collection, we as the Epidemiologic Architecture for Genes Linked to Environment (EAGLE) study accessed the National Health and Nutrition Examination Surveys (NHANES) linked to DNA samples (“Genetic NHANES”) from NHANES III and NHANES 1999–2002. NHANES are population-based cross-sectional surveys conducted by the National Center for Health Statistics at the Centers for Disease Control and Prevention. Genome-wide genetic data is not yet available in NHANES, and current data use agreements prohibit the generation of GWAS-level data in NHANES samples due issues in maintaining confidentiality among other ethical concerns. To date, only hundreds of single nucleotide polymorphisms (SNPs) genotyped in a variety of candidate genes are available for analysis in NHANES. We performed identity-by-descent (IBD) estimates in three self-identified subpopulations of Genetic NHANES (non-Hispanic white, non- Hispanic black, and Mexican American) using PLINK software to identify potential familial relationships from presumed unrelated subjects. We then compared the PLINKidentified relationships to those identified by an alternative method implemented in *K*inship-based *IN*ference for Genome-wide association studies (KING). Overall, both methods identified familial relationships in NHANES III and NHANES 1999–2002 for all three subpopulations, but little concordance was observed between the two methods due in major part to the limited SNP data available in Genetic NHANES. Despite the lack of genome-wide data, our results suggest the presence of cryptic relatedness in this epidemiologic collection and highlight the limitations of restricted datasets such as NHANES in the context of modern day genetic epidemiology studies.

## Introduction

Epidemiologic cohorts are a valuable resource for genotype–phenotype studies given their large sample size, racial/ethnic diversity, and wealth of phenotypes, traits, and exposures. Though typically ascertained for specific phenotypes, the breadth of phenotypic and environmental variables makes these cohorts particularly well-suited for inclusion in secondary analyses. With the addition of genetic data such as genotypes, these epidemiologic cohorts are positioned for inclusion in genetic association or linkage studies. Some examples of well-characterized studies that have genetic data include the Framingham Heart Study (Splansky et al., 2007), Women’s Health Initiative (Anderson et al., 2003), and the Jackson Heart Study (Taylor, 2005).

Some epidemiologic cohorts specifically seek related individuals during the ascertainment process in order to study the heritability of certain traits in a similar genetic background or minimize certain environmental differences between individuals; for example, the Framingham Heart Study recruited participants from a Massachusetts town and subsequently enrolled their offspring in later phases of the study to identify factors that contribute to cardiovascular disease (Splansky et al., 2007), whereas the Marshfield Clinic Personalized Medicine Research Project participants are relatively ethnically homogenous and come from the Marshfield, Wisconsin area (McCarty et al., 2005). Others, such as the National Health and Nutrition Examination Surveys (NHANES), use an ascertainment process where multiple participants from a single household may be included without documentation of the relationship between those participants (Ezzati et al., 1992).

Genetic association studies are susceptible to confounding, through population substructure and cryptic relatedness (Astle and Balding, 2009). Population substructure and cryptic relatedness both result from underlying relatedness between individuals in a population that is greater than the amount expected in a freely mating population. Distant relatedness, relatedness that occurs on a macro level such as racial/ethnic or genetic ancestry is a known confounder in association studies, and multiple methods exist for its identification and adjustment in analysis (Liu et al., 2013). Cryptic relatedness implies unknown (undocumented) more recent relatedness and includes family relationships such as grandparent-grandchild and full sibling pairs. While known family structure is essential for genetic linkage or family based association studies, population-based association studies assume independent (unrelated) individuals. Cryptic relatedness in studies that assume independent individuals results in inflated effect size estimates and possible false positive associations; thus, adjustment for familial relationships within population-based studies is considered necessary.

Several methods have been developed to infer familial relationships primarily for linkage studies, including Graphical Representation of Relationship errors (GRR; Abecasis et al., 2001) and multiple hidden Markov model (HMM) programs (Boehnke and Cox, 1997; McPeek and Sun, 2000). Contemporary linkage studies consist of pedigrees of varying size and thousands of diallelic markers, and methods such as GRR are routinely applied to these data to identify errors in pedigree assignment. While existing methods such as GRR could be applied to unrelated samples to identify cryptic relatedness, the performance of this simple clustering method is dependent both on the number of samples genotyped and the number and frequency of the markers genotyped. The need for consistent identification of cryptic relatedness coupled with the availability of dense marker panels led to the development of algorithms for genome-wide association studies which typically consist of thousands of individuals (either unrelated or as pedigrees) and hundreds of thousands to millions of diallelic markers.

One such GWAS-developed algorithm is implemented in PLINK, a widely used software program for genetic association studies that calculates identity-by-descent (IBD) using a combination of identity-by-state (IBS) between individuals and allele frequency at each single nucleotide polymorphism (SNP), assuming Hardy–Weinberg Equilibrium (Purcell et al., 2007). At a given locus, two individuals carrying the same allele are said to be IBD if the alleles arose from the same ancestral allele; if the same alleles are not the product of the same ancestral allele, they are said to be IBS. An alternate method, *K*inship-based *IN*ference for *G*enome-wide association studies (KING) was recently developed to infer relationships using kinship coefficients (Manichaikul et al., 2010). A kinship coefficient is the probability that at a specific locus, the allele picked at random in two individuals is IBD (Lange and Sinsheimer, 1992). Both of these methods assume large numbers of SNPs to calculate relatedness between pairs of individuals and may not accurately assign distant relationships when a small number of SNPs is used. In both simulated and actual GWAS-level data, KING is computationally faster than PLINK, and its framework is more flexible allowing for small sample sizes and population heterogeneity.

Most but not all epidemiologic cohorts or cross-sectional studies available for genetic association studies have GWAS-level data available on all or a fraction of the participants available. For these GWAS-less studies, basic quality control for genetic association studies such as the identification of cryptic relatedness or population substructure can be a challenge. One such study that routinely faces this challenge is the large, population-based NHANES. DNA samples were obtained from NHANES participants between 1991 and 1994 (NHANES III) and NHANES 1999–2002, and Genetic NHANES consists of several racial/ethnic subgroups: non-Hispanic whites (*n* = 6,634), non-Hispanic blacks (*n* = 3,458), and Mexican Americans (*n* = 3,950). Genetic NHANES has been genotyped for hundreds of SNPs (range *n* = 364–784) mostly selected to replicate and generalize genome-wide association study findings in diverse populations as part of the Epidemiologic Architecture for Genes Linked to Environment (EAGLE) study, a study site of the larger Population Architecture using Genomics and Epidemiology (PAGE) I study (Matise et al., 2011).

National Health and Nutrition Examination Survey participants are recruited by household; however, familial relationships are not consistently included in the data collection process. To understand the extent of gross cryptic relatedness, we inferred familial relationships using PLINK and KING, both developed for genetic association study settings involving large samples of presumably unrelated participants. Despite limited resolution, we identified cryptic relatedness in these large cross-sectional surveys using both of these methods and call attention to the potential for hidden familial relationships in epidemiologic cohorts accessed for genetic association studies.

## Materials and Methods

### Study Population

The National Health and Nutrition Examination Surveys are population-based surveys conducted across the U. S. by the National Center for Health Statistics (NCHS) at the Centers for Disease Control and Prevention (CDC). NHANES began in the 1960s; the Third NHANES (NHANES III) was performed from 1988 to 1994 and included 33,994 participants. From 1999, NHANES surveys have been performed continuously. NHANES 1999–2002 included 25,316 participants. NHANES capture self-described race/ethnicity on all participants. Oversampling of Mexican Americans, non-Hispanic blacks, children, and the elderly were performed to create nationally representative samples. DNA is available for 7,159 participants over age 12 in NHANES III (non-Hispanic white, *n* = 2,631; non-Hispanic black, *n* = 2,108; Mexican American, *n* = 2,073; other, *n* = 348) and for 7,839 participants (non-Hispanic white, *n* = 4003; non-Hispanic black, *n* = 1350; Mexican American, *n* = 1877; other Hispanic, *n* = 418; other race including multi-racial, *n* = 191) in NHANES 1999–2002 (Crawford et al., unpublished). Written informed consent was obtained from all participants. The CDC Ethics Review Board reviewed the present study, and the de-identified NHANES data were considered non-human subjects research by the Vanderbilt University Institutional Review Board.

### Genotyping

Single nucleotide polymorphisms were selected for either candidate gene studies (Crawford et al., 2006, 2010, 2015; Limdi et al., 2010; Dumitrescu et al., 2011c; Jeff et al., 2012, 2015) or for replication/generalization studies (Dumitrescu et al., 2011a,b; Haiman et al., 2012; Murabito et al., 2012; Spencer et al., 2012; Wassel et al., 2012; Carty et al., 2013; Fesinmeyer et al., 2013a,b; Goodloe et al., 2013; Zhang et al., 2013a,b; Crawford et al., 2014; Jeff et al., 2014; Mitchell et al., 2014; Restrepo et al., 2014, 2015; Villegas et al., 2014), the majority of the latter as part of the Population Architecture using Genetics and Epidemiology (PAGE) I Study (Matise et al., 2011). Genotypes were generated using Taqman, Illumina BeadXpress, or Sequenom in the Vanderbilt University’s Center for Human Genetics Research DNA Resources Core and the Open Wet Laboratory (OWL) Resource (Matise et al., 2011; Crawford et al., unpublished) or were accessed from existing data in the NHANES Genetic Database (Chu et al., 2009; Keebler et al., 2009). All genetic variants available in NHANES including those accessed here can be found on the CDC website (www.nhgeneticvariant.com, http://www.cdc.gov/nchs/nhanes/biospecimens/DNAspecimens.htm).

### Statistical Analysis

Participants were stratified by self-reported race/ethnicity in both NHANES datasets; IBD was estimated in non-Hispanic white, non-Hispanic black, and Mexican American subgroups. SNPs were excluded from the analysis if, within a given NHANES race/ethnicity, they were significantly out of Hardy–Weinberg Equilibrium (HWE; *p* < 0.001). To obtain a set of independent SNPs, we further excluded SNPs that were in high linkage disequilibrium (LD) with each other (*r*^2^ > 0.60) using the –indep command in PLINK. Two different programs, PLINK (Purcell et al., 2007) and KING (Manichaikul et al., 2010), were used to calculate IBD within each NHANES subgroup.

The –genome command was used to calculate the IBD estimates using PLINK. We pre-specified the classification scheme for potential familial relationships based on ranges of *z*-scores and π-hat scores (proportion IBD) for PLINK (**Table 1**). The –kinship command with additional parameters of –homo and –show-IBD were used to calculate IBD estimates using KING. Kinship coefficient ranges were used to identify potential familial relationships in KING (**Table 1**). Further classification of first degree relationships from KING were obtained by evaluating the IBD0 values; parent/child relationships will have IBD0 = 0 except for instances of genotyping error. We compared the ability of the software programs to identify cryptic relatedness within NHANES racial/ethnic groups using less than 1,000 SNPs. Due to the data use agreement with the CDC, any participant counts < 5 are suppressed for confidentiality concerns.

**Table 1:** PLINK and KING variable ranges for calculating familial relationships.

		PLINK				KING
			
Potential relationship		*z*0	*z*1	*z*2	π-hat	Kinship coefficient
Duplicate/identical twin		0-0.10	0-0.10	0.90-1.00	>0.95	>0.354
First degree	Parent/child	0	0.80-1.00	0-0.20	0.50-0.60	0.177-0.354
	Full siblings	0.15-0.35	0.40-0.60	0.15-0.35	0.40-0.60	0.177-0.354
Second degree		0.45-0.55	0.45-0.55	0	0.23-0.27	0.0884-0.177
Third degree		0.70-0.80	0.20-0.30	0	0.10-0.15	0.0442-0.0884


*Shown are the PLINK ox*z*-score and oxπ-hat ranges and the KING kinship coefficient ranges for calculating familial relationships using each software program. Kinship coefficient ranges for parent/child and full sibling in KING are equal as there is no further discrimination of familial status for first degree relationships using the kinship coefficient with that software. Parent/child and full sibling relationships in KING were discriminated using both kinship coefficient and IBD0 values as described in the manuscript.*

## Results

We calculated IBD estimates and potential familial relationships in NHANES III (*n* = 6,811) and NHANES 1999–2002 (*n* = 7,230) non-Hispanic whites, non-Hispanic blacks, and Mexican Americans using PLINK and KING software. In both NHANES III and NHANES 1999–2002, the majority of the participants were women (NHANES III, female: 56.67%; NHANES 1999–2002, female: 51.79%; **Table 2**). In each NHANES dataset, non-Hispanic whites comprised the largest subgroup (**Table 2**). The median age in NHANES III was lower (median age = 38.00) than in NHANES 1999–2002 (median age = 47.00; **Table 2**). Household size ranged from 1 to 10 participants in NHANES III and 1–7 participants in NHANES 1999–2002. In both surveys, two-person households were the most common overall, though there were differences by subgroup (**Table 2**). Mexican Americans had the largest median household size (*n* = 4) and widest range for household size in both NHANES (NHANES III: 1–10; NHANES 1999–2002: 1–7; **Table 2**).

**Table 2:** Demographics of Genetic National Health and Nutrition Examination Surveys (NHANES).

	NHANES III		NHANES 1999–2002	
		
	Participants	SNPs	Participants	SNPs
***N*, by subgroup**				
NHW	2,631	721	4,003	364
NHB	2,108	691	1,350	367
MEX	2,073	784	1,877	380
**% female, total**	56.67		51.79	
% female				
NHW	60.00		51.14	
NHB	57.45		52.44	
MEX	50.60		51.73	
**Median age in years (range), total**	38.00 (12–90)		47.00 (20–85)	
Median age (range)				
NHW	(12–90)		(20–85)	
NHB	(12–90)		(20–85)	
MEX	(12–90)		(20–85)	
**Median household size (range), total**	2.00 (1–10)		2.00 (1–7)	
Median household size (range)				
NHW	2.00 (1–8)		2.00 (1–7)	
NHB	3.00 (1–10)		3.00 (1–7)	
MEX	4.00 (1–10)		4.00 (1–7)	


*Data shown are participants from the National Health and Nutrition Examination Survey (NHANES) III and NHANES 1999–2002 and SNPs used for cryptic relatedness analysis. Data are counts unless otherwise specified. The number of SNPs shown in the table represents the SNPs available after removing SNPs out of Hardy–Weinberg Equilibrium and LD pruning. SNPs, single nucleotide polymorphisms; NHW, non-Hispanic white; NHB, non-Hispanic black; MEX, Mexican American.*

**FIGURE 1:**
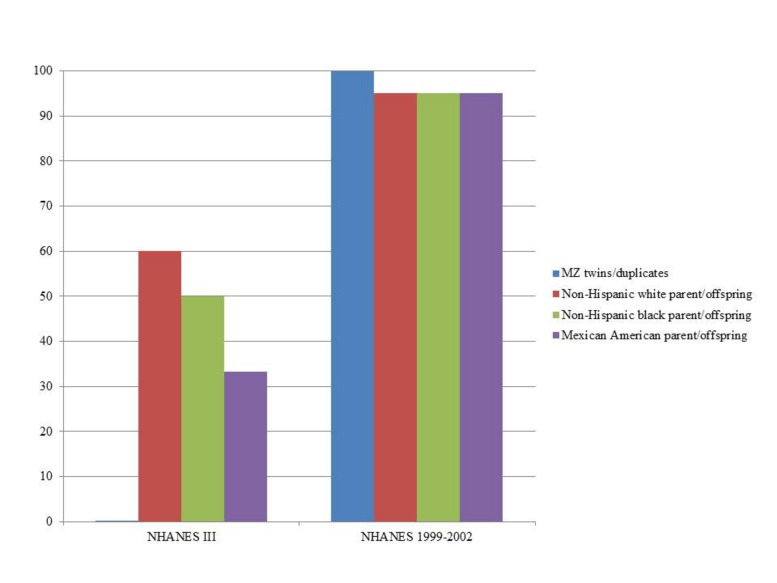
**Percent concordance of familial relationships identified by KING compared with PLINK by survey.** KING relationships were considered concordant if also identified by PLINK (expressed as percent on the y-axis). We only consider close relationships here (monozygotic or MZ twins, duplicate samples, and parent-offspring) in estimating concordance given the limited SNP data and the lack of resolution expected with these data. The data are displayed by Genetic NHANES (x-axis) and stratified by estimated familial relationship and race/ethnicity.

In NHANES III, PLINK calculated more potential familial relationships than KING in all subgroups. We identified few instances of potential duplicate samples/monozygotic (MZ) twins (**Table 3**) using either program; however, we observed no concordance between the potential duplicates/MZ twin pairs (**Figure 1**). A number of first degree relationships (parent/offspring and full sibling) were calculated with both methods. PLINK calculated the greatest number of potential first degree relationships in non-Hispanic whites (*n* = 1,864) and the majority of these relationships were parent/offspring (*n* = 1,843), while Mexican Americans full sibling pairs were the most common first degree relationship calculated with KING (*n* = 194; **Table 3**). Across all subgroups, most first degree relationships were categorized by PLINK as parent/offspring and by KING as full sibling (**Table 3**). The greatest number of second degree and third degree relationships was observed in non-Hispanic blacks using PLINK (*n* = 237,154) and in non-Hispanic whites using KING (*n* = 162,609; **Table 3**). We found little concordance (<20% same relationship identified for a given participant pair) between the methods to identify the same participants’ relationships, though 60% of non-Hispanic white parent/offspring pairs calculated with KING were classified as parent/offspring pairs with PLINK (**Figure 1**). We observed similar concordance/discordance among the results for non-Hispanic black parent/offspring pairs (50% concordant) and Mexican American parent/offspring pairs (33.33% concordant; **Figure 1**). Within the discordant results in each subgroup, a number of each potential familial relationship identified through one method was classified as a different familial relationship using the other method. Where the methods yielded a different potential familial relationship for a given participant pair, we found PLINK calculated the pair as more related (e.g., KING calculated third degree relationships classified as second degree or first degree in PLINK). This observation held across all subgroups and familial relationships, except second degree relationships in non-Hispanic blacks and Mexican Americans, where KING-calculated second degrees were more frequently classified as PLINK third degrees than any other relationship. Broadly, PLINK and KING identified numerous potential first, second, and third degree relationships in NHANES III; however, there was little concordance between the results.

**Table 3:** Probable familial relationships identified in NHANES III.

NHANES III		Duplicate/MZ twin	First degree		Second degree	Third degree
					
			Parent-offspring	Full-siblings		
NHW	PLINK	<5	1843	21	36,602	182,856
	KING	<5	5	79	12,469	150,140
NHB	PLINK	<5	391	47	39,955	197,199
	KING	<5	<5	131	11,004	142,412
MEX	PLINK	<5	329	97	23,697	170,430
	KING	<5	12	194	7,338	130,040


*The numbers represent counts of individual pairs such that an individual can be in multiple pairings. Data are counts where possible [CDC data use agreement requires any count(s) <5 to be suppressed for confidentiality concerns]. NHW, non-Hispanic white; NHB, non-Hispanic black; MEX, Mexican American; MZ, monozygotic.*

In NHANES 1999–2002, we again observed PLINK calculated a greater number of potential familial relationships than KING for non-Hispanic whites and Mexican Americans; KING calculated a greater number of familial relationships in non-Hispanic blacks (**Table 4**). There were few instances of MZ twins/duplicate samples among the three subgroups and the participant pairs were concordant between the two methods (**Table 4**; **Figure 1**). Fewer overall first degree relationships were calculated in NHANES 1999–2002 than in NHANES III (**Tables 3** and **4**). Similar to the trends observed in NHANES III, KING identified fewer instances of first degree relationships than PLINK (**Table 4**). PLINK calculated the greatest number of first degree relationships in non-Hispanic blacks (*n* = 498) and the majority of these were parent/offspring (*n* = 493), whereas Mexican American full sibling relationships were the most numerous relationship calculated by KING (*n* = 140; **Table 4**). The greatest number of second and third degree relationships were observed in non-Hispanic whites (*n* = 282,512) using PLINK, and in non-Hispanic blacks (*n* = 167,925) using KING (**Table 4**). As with the NHANES III results, we found little concordance (<25%) between the participant pairs identified within each potential familial relationship with the exception of parent/offspring pairs across the three subgroups, in which >95% of parent/offspring pairs identified with KING were also identified with PLINK (**Figure 1**). In addition, we observed a similar trend within discordant pairs, where PLINK calculated the pair as more related than with KING, with the exception of non-Hispanic white second degree relationships which were more often classified as third degree relationships in PLINK. Overall, we identified potential cryptic relatedness in NHANES 1999–2002 using PLINK and KING, though concordance between the two methods was generally lacking.

**Table 4:** Probable familial relationships identified in NHANES 1999–2002.

NHANES 1999–2002		Duplicate/MZ twin	First degree		Second degree	Third degree
					
			Parent-offspring	Full-siblings		
NHW	PLINK	<5	274	<5	59,792	222,720
	KING	<5	7	37	12,029	116,259
NHB	PLINK	<5	493	5	29,575	77,841
	KING	<5	41	126	23,695	144,230
MEX	PLINK	<5	180	23	40,559	160,322
	KING	<5	31	140	15,397	126,031


*The numbers represent counts of individual pairs such that an individual can be in multiple pairings. Data are counts where possible (CDC data use agreement requires any count(s) <5 to be suppressed for confidentiality concerns). NHW, non-Hispanic white; NHB, non-Hispanic black; MEX, Mexican American; MZ, monozygotic.*

## Discussion

Cryptic relatedness is a source of confounding in population-based genetic association studies. Population stratification due to distant ancestry is typically accounted for in genetic association studies; however, more recent shared ancestry may not be considered. Though beneficial for epidemiologic, linkage, and family based association studies, close relatedness (first and second degree) may lead to spurious results and/or inflated effect estimates in population-based association studies where an underlying assumption is that the individuals are unrelated. Additionally, specifically testing the influence of multiple interactions on heritability estimates requires known relatedness between individuals (Patel et al., 2013). Epidemiologic cohorts and cross-sectional surveys that inconsistently collect pedigree data on their participants are particularly at risk for confounding due to cryptic relatedness.

We estimated likely familial relationships in NHANES III and NHANES 1999–2002 using ∼730 and ∼370 SNPs, respectively, and two methods, PLINK and KING. Using PLINK, which estimates IBD by calculating IBS and allele frequencies at each SNP, we observed many potential first degree relationships in NHANES III across the three population subgroups. The majority of these first degree relationships in PLINK were likely parent/offspring pairs. In NHANES 1999–2002, we observed fewer likely first degree relationships than in NHANES III. Thousands of potential second and third degree relationships were calculated using PLINK in both NHANES III and NHANES 1999–2002. Using KING, which estimates IBD using kinship coefficients, we observed far fewer first degree relationships in NHANES III and NHANES 1999–2002 than with PLINK. The majority of the first degree relationships calculated by KING were full sibling pairs, in contrast to the parent/offspring majority identified with PLINK. In NHANES 1999–2002, the majority of first degree relationships were full sibling pairs. We identified numerous potential second and third degree relationships using KING. Using both PLINK and KING, which calculate familial relationships with different statistical methods, we observed relatedness in NHANES III and NHANES 1999–2002 across non-Hispanic white, non-Hispanic black, and Mexican American subgroups.

An earlier study evaluated familial relationships in NHANES III using short tandem repeats (STRs) at fifteen DNA loci used in forensic investigations (Identifiler^®^) and found evidence of first and second degree relationships (Katki et al., 2010). Studies suggest that approximately 50 SNPs yield the same relationship discrimination as 13–15 STRs (Butler, 2007), and paternity results obtained by STR and SNP analysis are generally comparable (Dario et al., 2009). In acknowledgment of the lack of self-reported pedigree information for NHANES III participants, Katki et al. (2010) calculated the likely relationships for participants living in multi-person households with an exact method and an IBS method. Both methods were generally consistent in identifying first degree relationship pairs, but there was greater variation in the classification of likely second degree and first cousin relationships. It was further observed that the STRs in the Identifiler test were not informative enough to accurately discriminate between second degree, third degree, and unrelated participants (Katki et al., 2010). Using information not available to our study, Katki et al. (2010) also compared the likely familial relationships with the ages of the participants; they found only 5% of the parent-offspring pairs had age differences less than 16 years and 9% of sibling pairs with age differences greater than 25 years, suggesting few of their proposed first degree relationships were misclassified. However, their method may have underestimated the true level of relatedness in NHANES III by restricting potential familial relationships to participants from the same multi-person household; given the NHANES ascertainment process, it is likely additional family relationships exist, such as siblings or second and third degree relationships, within a given geographic region that may be present in the NHANES datasets.

In general, our results are consistent with those of Katki et al. (2010). We observed evidence of cryptic relatedness in NHANES III using both PLINK and KING methods. Similar to the counts observed in Katki et al. (2010), our PLINK results uncovered a high number of parent-offspring relationships. In contrast, our KING results calculated fewer first degree relationships and classified the majority of them as full sibling pairs. The similarities between our PLINK-calculated likely relationships and those obtained by Katki et al. (2010) may be due to PLINK’s method of calculating IBD using IBS and allele frequencies, though notably, in Katki et al. (2010), the exact method classification and IBS method were nearly identical. Our study is the first, to our knowledge, to consider cryptic relatedness in NHANES 1999–2002; therefore, no comparison to other studies could be drawn.

Despite the general agreement of our study with the previous Katki et al. (2010) work demonstrating cryptic relatedness in NHANES III and the results of the present study for NHANES 1999–2002, we found no evidence to suggest cryptic relatedness has yet led to inflated effect sizes or spurious positive associations in published studies accessing these NHANES datasets. Most of the published studies that have used the genetic NHANES datasets have been replication studies or generalization of prior findings to diverse populations (Crawford et al., unpublished). Formal meta-analysis of genetic NHANES association data with other epidemiologic cohorts generally does not reveal significant genetic heterogeneity or differences in genetic effect sizes (Dumitrescu et al., 2011b; Haiman et al., 2012; Carty et al., 2013; Fesinmeyer et al., 2013b; Jeff et al., 2014; Restrepo et al., 2014). Future studies that access these datasets to identify novel genetic variants should consider the potential for false positives and/or inflated effect sizes in their results and verify allele frequencies are comparable to published data.

A limitation to our study is the small number of SNPs used to identify likely familial relationships. Also, the SNPs in NHANES III do not necessarily overlap with SNPs in NHANES 1999–2002, resulting in different SNP sets with different polymorphism information content. These differences may account in part for the observation that PLINK results were less concordant with KING results in NHANES III compared with NHANES 1999–2002 despite the greater number of SNPs available for analysis. In general, PLINK, KING, and other software programs that calculate cryptic relatedness, require large numbers of SNPs (typically GWAS-level data) to calculate IBD; the more markers are used in the calculation, the greater the stability and accuracy of the IBD estimates (Marchani et al., 2009), and the greater the confidence in the estimated familial relationships. Using fewer than the thousands of SNPs expected by these programs may have resulted in inflation of IBD estimates in our study and led to the discordant results between the two methods that we observed. A proportion of the discordant results may also be due to the fact that PLINK requires independent SNPs whereas KING does not. We used the same SNP set for both programs to make direct comparisons, but this restriction to independent SNPs coupled with the already limited number of SNPs available in Genetic NHANES may have compounded the discordance observed between the two methods.

Most epidemiologic studies with DNA have GWAS data, allowing investigators to more accurately assess the level of relatedness in the dataset, contrasting with the problem of assessing cryptic relatedness in genotype-limited datasets such as NHANES. Per CDC data use agreements, GWAS-level genotyping is not permitted at this time by investigators outside of CDC or without a CDC contract. While there are multiple programs for accurately estimating IBD given thousands of SNPs, there are relatively few options for epidemiologic collections with hundreds or fewer genotyped SNPs. Recently, a new R package, CrypticIBDCheck, was published demonstrating the ability to calculate IBD with as few as 60 candidate genes (∼300 SNPs) which have not been LD pruned for independence (Nembot-Simo et al., 2013). Future studies of Genetic NHANES should evaluate this and emerging tools to identify cryptic relatedness and properly adjust for its potential impact on downstream genetic association studies.

Another limitation of the current study is the lack of pedigree data available in NHANES, which unlike simulated data with known pedigree structures used to originally compare PLINK and KING (Manichaikul et al., 2010), prohibits the full evaluation of any method used to identify cryptic relatedness. Both PLINK and KING identified familial relationships in Genetic NHANES, but there was little concordance with respect to the number and type of familial relationships between the two methods. Under this scenario, alternative approaches to adjusting for cryptic relatedness in downstream analyses (such as generalized estimating equations to account for correlated data (Tregouet et al., 1997) may be more appropriate. Adjustment for correlated data has the additional advantage of preserving sample size and power compared with the usual practice of removing individuals from datasets after relationships have been identified. However, like PLINK and KING, methods such as mixed linear models (Yang et al., 2014) work best with genome-wide as opposed to sparse candidate gene data.

In summary, we have estimated familial relationships in Genetic NHANES, an epidemiologic cross-sectional study with sparse candidate gene SNPs available for genetic association studies. We implemented the commonly used PLINK and compared these familial relationships to those estimated by a second method implemented in KING. Familial relationships were identified in Genetic NHANES using both methods, but little concordance was observed between the methods. In absence of pedigree data, it is not possible to determine which of the two methods is more accurate in estimating familial relationships in this collection of cross-sectional surveys. In absence of GWAS-level data, basic quality control will continue to be a challenge for NHANES and similar epidemiologic collections. Further research is needed to identify alternate approaches to identify cryptic relatedness using sparse SNP data such as those available in Genetic NHANES.

## Author Contributions

The listed authors provided substantial contributions to the conception or design of the work (JM, DC), the acquisition (DC), analysis (RG, KB-G), or interpretation of the data (JM, RG, KB-G) for the work; drafted the work (JM) or revised it critically for important intellectual context (RG, KB-G, DC); gave final approval of the version to be published (JM, RG, KB-G, DC); and agreed to be accountable for all aspects of the work in ensuring that questions related to accuracy or integrity of any part of the work are appropriately investigated and resolved (JM, RG, KB-G, DC).

## Conflict of Interest Statement

The authors declare that the research was conducted in the absence of any commercial or financial relationships that could be construed as a potential conflict of interest.

## References

[B1] Abecasis G. R. Cherny S. S. Cookson W. O. C. Cardon L. R. (2001). GRR: graphical representation of relationship errors. *Bioinformatics* 17 742–743. 11524377 10.1093/bioinformatics/17.8.742

[B2] Anderson G. L. Manson J. Wallace R. Lund B. Hall D. Davis S. (2003). Implementation of the women’s health initiative study design. *Ann. Epidemiol.* 13 S5–S17. 10.1016/S1047-2797(03)00043-7 14575938

[B3] Astle W. Balding D. J. (2009). Population structure and cryptic relatedness in genetic association studies. *Stat. Sci.* 24 451–471. 10.1214/09-STS307

[B4] Boehnke M. Cox N. J. (1997). Accurate inference of relationships in sib-pair linkage studies. *Am. J. Hum. Genet.* 61 423–429. 10.1086/514862 9311748 PMC1715905

[B5] Butler J. M. (2007). Short tandem repeat typing technologies used in human identify testing. *Biotechniques* 43 II–V 10.2144/000112582 18019344

[B6] Carty C. L. Spencer K. L. Setiawan V. W. Fernandez-Rhodes L. Malinowski J. Buyske S. (2013). Replication of genetic loci for ages at menarche and menopause in the multi-ethnic Population Architecture using Genomics and Epidemiology (PAGE) Study. *Hum. Reprod.* 28 1695–1706. 10.1093/humrep/det071 23508249 PMC3657124

[B7] Chu A. Y. Parekh R. S. Astor B. C. Coresh J. Berthier-Schaad Y. Smith M. W. (2009). Association of *APOE* polymorphism with chronic kidney disease in a nationally representative sample: a Third National Health and Nutrition Examination Survey (NHANES III) *Genetic Study*. *BMC Med. Genet.* 10:108 10.1186/1471-2350-10-108 PMC2770999 19852818

[B8] Crawford D. C. Brown-Gentry K. Rieder M. J. (2010). VKORC1 common variation and bone mineral density in the Third National Health and Nutrition Examination Survey. *PLoS ONE* 5:e15088 10.1371/journal.pone.0015088 PMC3001474 21179439

[B9] Crawford D. C. Brown-Gentry K. Rieder M. J. (2015). Measures of exposure impact genetic association studies: an example in vitamin K levels and VKORC1. *Pac. Symp. Biocomput.* 2015 161–170. 25592578 PMC4299921

[B10] Crawford D. C. Dumitrescu L. Goodloe R. Brown-Gentry K. Boston J. McClellan B. (2014). Rare variant APOC3 R19X is associated with cardio-protective profiles in a diverse population-base survey as part of the Epidemiologic Architecture for Genes Linked to Environment (EAGLE) Study. *Circ. Cardiovasc. Genet.* 7 848–853. 10.1161/CIRCGENETICS.113.000369 25363704 PMC4305446

[B11] Crawford D. C. Sanders C. L. Qin X. Smith J. D. Shephard C. Wong M. (2006). Genetic variation is associated with C-reactive protein levels in the Third National Health and Nutrition Examination Survey. *Circulation* 114 2458–2465. 10.1161/CIRCULATIONAHA.106.615740 17101857

[B12] Dario P. Ribeiro T. Espinheira R. Geada H. (2009). SNPs in paternity investigation: the simple future. *Forensic Sci. Int. Genet. Suppl. Ser.* 2 127–128.

[B13] Dumitrescu L. Brown-Gentry K. Goodloe R. Glenn K. Yang W. Kornegay N. (2011a). Evidence for age as a modifier of genetic associations for lipid levels. *Ann. Hum. Genet.* 75 589–597. 10.1111/j.1469-1809.2011.00664.x 21777205 PMC3155612

[B14] Dumitrescu L. Carty C. L. Taylor K. Schumacher F. R. Hindorff L. A. Ambite J.-L. (2011b). Genetic determinants of lipid traits in diverse populations from the population architecture using genomics and epidemiology (PAGE) study. *PLoS Genet.* 7:e1002138 10.1371/journal.pgen.1002138 PMC3128106 21738485

[B15] Dumitrescu L. Glenn K. Brown-Gentry K. Shephard C. Wong M. Rieder M. J. (2011c). Variation in LPA is associated with lp(a) levels in three populations from the Third National Health and Nutrition Examination Survey. *PLoS ONE* 6:e16604 10.1371/journal.pone.0016604 PMC3030597 21305047

[B16] Ezzati T. M. Massey J. T. Waksberg J. Chu A. Maurer K. R. (1992). Sample design: Third National Health and Nutrition Examination Survey. *Vital Health Stat.* 2 1–35. 1413563

[B17] Fesinmeyer M. D. Meigs J. B. North K. E. Schumacher F. R. Buzkova P. Franceschini N. (2013a). Genetic variants associated with fasting glucose and insulin concentrations in an ethnically diverse population: results from the Population Architecture using Genomics and Epidemiology (PAGE) Study. *BMC Med. Genet.* 14:98 10.1186/1471-2350-14-98 PMC3849560 24063630

[B18] Fesinmeyer M. D. North K. E. Ritchie M. D. Lim U. Franceschini N. Wilkens L. R. (2013b). Genetic risk factors for body mass index and obesity in an ethnically diverse population: results from the Population Architecture using Genomics and Epidemiology (PAGE) Study. *Obesity (Silver Spring)* 21 835–846. 10.1002/oby.20268 23712987 PMC3482415

[B19] Goodloe R. Brown-Gentry K. Gillani N. Jin H. Mayo P. Allen M. (2013). Lipid trait-associated genetic variation is associated with gallstone disease in the diverse Third National Health and Nutrition Examination Survey (NHANES III). *BMC Med. Genet.* 14:120 10.1186/1471-2350-14-120 PMC3870971 24256507

[B20] Haiman C. A. Fesinmeyer M. Spencer K. L. Buzkova P. Voruganti V. S. Wan P. (2012). Consistent direction of effect for established T2D risk variants across populations: the Population Architecture using Genomics and Epidemiology (PAGE) consortium. *Diabetes Metab. Res. Rev.* 61 1642–1647. 10.2337/db11-1296 PMC3357304 22474029

[B21] Jeff J. M. Brown-Gentry K. Crawford D. C. (2012). Replication and characterisation of genetic variants in the fibrinogen gene cluster with plasma fibrinogen levels and haematological traits in the Third National Health and Nutrition Examination Survey. *Thromb. Haemost.* 107 458–467. 10.1160/TH11-07-0497 22273812 PMC3989929

[B22] Jeff J. M. Brown-Gentry K. Crawford D. C. (2015). Identification of genetic modifiers within the fibrinogen gene cluster for fibrinogen levels in three ethnically diverse populations. *Pac. Symp. Biocomput.* 2015 219–230. 25592583 PMC4357227

[B23] Jeff J. M. Brown-Gentry K. Goodloe R. Ritchie M. D. Denny J. C. Kho A. N. (2014). Replication of SCN5A associations with electrocardiographic traits in African Americans from clinical and epidemiologic studies. *Evol. Comput. Mach. Learn. Data Min. Bioinform.* 2014 939–951. 25590050 10.1007/978-3-662-45523-4_76 PMC4290789

[B24] Katki H. A. Sanders C. L. Graubard B. I. Bergen A. W. (2010). Using DNA fingerprints to infer familial relationships wihtin NHANES III households. *J. Am. Stat. Assoc.* 105 552–563. 10.1198/jasa.2010.ap09258 20664713 PMC2909633

[B25] Keebler M. E. Sanders C. L. Surti A. Guiducci C. Burtt N. P. Kathiresan S. (2009). Association of blood lipids with common DNA sequence variants at 19 genetic loci in the multiethnic united states National Health and Nutrition Examination Survey III / CLINICAL PERSPECTIVE. *Circ. Cardiovasc. Genet.* 2 238–243. 20031591 10.1161/CIRCGENETICS.108.829473 PMC3561731

[B26] Lange K. Sinsheimer J. S. (1992). Calculation of genetic identify coefficients. *Ann. Hum. Genet.* 56 339–346. 10.1111/j.1469-1809.1992.tb01162.x 1492748

[B27] Limdi N. A. Wadelius M. Cavallari L. Eriksson N. Crawford D. C. Lee M. T. (2010). Warfarin pharmacogenetics: a single VKORC1 polymorphism is predictive of dose across 3 racial groups. *Blood* 115 3827–3834. 10.1182/blood-2009-12-255992 20203262 PMC2865873

[B28] Liu Y. Nyunoya T. Leng S. Belinsky S. Tesfaigzi Y. Bruse S. (2013). Softwares and methods for estimating genetic ancestry in human populations. *Hum. Genomics* 7 1 10.1186/1479-7364-7-1 PMC3542037 23289408

[B29] Manichaikul A. Mychaleckyj J. C. Rich S. S. Daly K. Sale M. Chen W. M. (2010). Robust relationship inference in genome-wide association studies. *Bioinformatics* 26 2867–2873. 10.1093/bioinformatics/btq559 20926424 PMC3025716

[B30] Marchani E. E. Di Y. Choi Y. Cheung C. Su M. Boehm F. (2009). Constrasting identify-by-descent estimators, association studies, and linkage analyses using the Framingham Heart Study data. *BMC Proc.* 3:S102 10.1186/1753-6561-3-s7-s102 PMC2795873 20017966

[B31] Matise T. C. Ambite J. L. Buyske S. Carlson C. S. Cole S. A. Crawford D. C. (2011). The next PAGE in understanding complex traits: design for the analysis of Population Architecture using Genetics and Epidemiology (PAGE) study. *Am. J. Epidemiol.* 174 849–859. 10.1093/aje/kwr160 21836165 PMC3176830

[B32] McCarty C. A. Wilke R. A. Giampietro P. F. Wesbrook S. D. Caldwell M. D. (2005). Marshfield Clinic Personalized Medicine Research Project (PMRP): design, methods and recruitment for a large population-based biobank. *Pers. Med.* 2 49–79. 10.1517/17410541.2.1.49 29793241

[B33] McPeek M. S. Sun L. (2000). Statistical tests for detection of misspecified relationships by use of genome-screen data. *Am. J. Hum. Genet.* 66 1076–1094. 10.1086/302800 10712219 PMC1288143

[B34] Mitchell S. L. Goodloe R. Brown-Gentry K. Pendergrass S. A. Murdock D. G. Crawford D. C. (2014). Characterization of mitochondrial haplogroups in a large population-based sample from the United States. *Hum. Genet.* 133 861–868. 10.1007/s00439-014-1421-9 24488180 PMC4113317

[B35] Murabito J. M. White C. C. Kavousi M. Sun Y. Y. Feitosa M. F. Nambi V. (2012). Association between chromosome 9p21 variants and the ankle-brachial index identified by a meta-analysis of 21 genome-wide association studies. *Circ. Cardiovasc. Genet.* 5 100–112. 10.1161/CIRCGENETICS.111.961292 22199011 PMC3303225

[B36] Nembot-Simo A. Graham J. McNeney B. (2013). CrypticIBDcheck: an R package for checking cryptic relatedness in nominally unrelated individuals. *Source Code Biol. Med.* 8 5 10.1186/1751-0473-8-5 PMC3764977 23384435

[B37] Patel C. Chen R. Kodama K. Ioannidis J. Butte A. (2013). Systematic identification of interaction effects between genome– and environment-wide associations in type 2 diabetes mellitus. *Hum. Genet.* 132 495–508. 10.1007/s00439-012-1258-z 23334806 PMC3625410

[B38] Purcell S. Neale B. Todd-Brown K. Thomas L. Ferreira M. A. Bender D. (2007). PLINK: a tool set for whole-genome association and population-based linkage analysis. *Am. J. Hum. Genet.* 81 559–575. 10.1086/519795 17701901 PMC1950838

[B39] Restrepo N. A. Mitchell S. L. Goodloe R. J. Murdock D. G. Haines J. L. Crawford D. C. (2015). Mitochondrial variation and the risk of age-related macular degeneration across diverse populations. *Pac. Symp. Biocomput.* 2015 243–254. 10.1142/9789814644730_0024 25592585 PMC4299880

[B40] Restrepo N. A. Spencer K. L. Goodloe R. Garrett T. A. Heiss G. Buzkova P. (2014). Genetic determinants of age-related macular degeneration in diverse populations from the PAGE study. *Invest. Ophthalmol. Vis. Sci.* 55 6839–6850. 10.1167/iovs.14-14246 25205864 PMC4214207

[B41] Spencer K. L. Glenn K. Brown-Gentry K. Haines J. L. Crawford D. C. (2012). Population differences in genetic risk for age-related macular degeneration and implications for genetic testing. *Arch. Ophthalmol.* 130 116–117. 10.1001/archopthalmol.2011.1370 22232482 PMC3326353

[B42] Splansky G. L. Corey D. Yang Q. Atwood L. D. Cupples L. A. Benjamin E. J. (2007). The Third Generation Cohort of the National Heart, Lung, and Blood Institute’s Framingham Heart Study: design, recruitment, and initial examination. *Am. J. Epidemiol.* 165 1328–1335. 17372189 10.1093/aje/kwm021

[B43] Taylor H. A. (2005). The Jackson Heart Study: an overview. *Ethn. Dis.* 15 1–3. 16317981

[B44] Tregouet D. A. Ducimetiere P. Tiret L. (1997). Testing association between candidate-gene markers and phentoype in related individuals, by use of estimating equations. *Am. J. Hum. Genet.* 61 189–199. 10.1086/513895 9246000 PMC1715871

[B45] Villegas R. Goodloe R. McClellan B. Boston J. Crawford D. (2014). Gene-carbohydrate and gene-fiber interactions and type 2 diabetes in diverse populations from the National Health and Nutrition Examination Surveys (NHANES) as part of the Epidemiologic Architecture for Genes Linked to Environment (EAGLE) study. *BMC Genet.* 15:69 10.1186/1471-2156-15-69 PMC4094781 24929251

[B46] Wassel C. L. Lamina C. Nambi V. Coassin S. Mukamal K. J. Ganesh S. K. (2012). Genetic determinants of the ankle-brachial index: a meta-analysis of a cardiovascular candidate gene 50K SNP panel in the candidate gene association resource (CARe) consortium. *Atherosclerosis* 222 138–147. 10.1016/j.atherosclerosis.2012.01.039 22361517 PMC3596171

[B47] Yang J. Zaitlen N. A. Goddard M. E. Visscher P. M. Price A. L. (2014). Advantages and pitfalls in the application of mixed-model association methods. *Nat. Genet.* 46 100–106. 10.1038/ng.2876 24473328 PMC3989144

[B48] Zhang L. Spencer K. L. Voruganti V. S. Jorgensen N. W. Fornage M. Best L. G. (2013a). Association of functional polymorphism rs2231142 (Q141K) in ABCG2 gene with serum uric acid and gout in four US populations: the Population Architecture using Genomics and Epidemiology (PAGE) Study. *Am. J. Epidemiol.* 177 923–932. 10.1093/aje/kws330 23552988 PMC4023295

[B49] Zhang L. Buzkova P. Wassel C. L. Roman M. J. North K. E. Crawford D. C. (2013b). Lack of associations of ten candidate coronary heart disease risk genetic variants and subclinical atherosclerosis in four U.S. populations: the Population Architecture using Genomics and Epidemiology (PAGE) study. *Atherosclerosis* 228 390–399. 10.1016/j.atherosclerosis.2013.02.038 23587283 PMC3717342

